# Toxic Epidermal Necrolysis in an HIV-Infected Patient

**DOI:** 10.7759/cureus.87982

**Published:** 2025-07-15

**Authors:** Hiebda Sofía M Martínez Jiménez

**Affiliations:** 1 Internal Medicine, Centro Médico Nacional Siglo XXI, Mexico City, MEX

**Keywords:** drug hypersensitivity, hiv infection, severe cutaneous adverse reactions, stevens–johnson syndrome, toxic epidermal necrolysis

## Abstract

Stevens-Johnson syndrome (SJS) and toxic epidermal necrolysis (TEN) are rare but potentially fatal cutaneous adverse drug reactions, mediated by type IV hypersensitivity mechanisms triggered by medications such as antibiotics, antiepileptics, allopurinol, and non-steroidal anti-inflammatory drugs (NSAIDs). Various risk factors, including human immunodeficiency virus (HIV) infection, increase susceptibility to these severe reactions. Early symptoms are often non-specific and may precede the characteristic skin and mucosal lesions. Diagnosis requires thorough clinical and laboratory evaluation, including causality assessment tools such as the Algorithm for Drug Causality in Epidermal Necrolysis (ALDEN). Management demands a multidisciplinary approach focusing on supportive care and infection prevention, with emerging evidence supporting immunomodulatory therapies like cyclosporine and combined intravenous immunoglobulin and corticosteroids. This case report describes a 36-year-old HIV-positive man who developed carbamazepine-induced TEN. The diagnosis of TEN was confirmed clinically and supported by a positive causality assessment using the ALDEN. The patient was managed with supportive care and immunomodulatory treatment with cyclosporine and systemic corticosteroids, leading to gradual re-epithelialization and clinical improvement. This case highlights the increased risk of severe cutaneous adverse reactions in HIV-positive patients. Early recognition and multidisciplinary management are essential for improved outcomes.

## Introduction

Stevens-Johnson syndrome (SJS) and toxic epidermal necrolysis (TEN) are rare, life-threatening cutaneous adverse drug reactions [1]. These conditions are classified as type IV hypersensitivity responses that are primarily triggered by medications. Over 80% of cases are linked to drug use, particularly antibiotics (notably sulfonamides), antiepileptic agents, allopurinol, and non-steroidal anti-inflammatory drugs [1]. Multiple risk factors contribute to the development of SJS/TEN, including underlying malignancies, human immunodeficiency virus (HIV) infection, genetic predisposition (including the presence of HLA-B*15:02 allele, which is strongly associated with carbamazepine-induced severe cutaneous adverse reactions), systemic lupus erythematosus, and radiation exposure [2].

A retrospective study conducted by Mittmann et al. within an HIV-positive population demonstrated an SJS/TEN incidence rate of approximately 1-2 cases per 1,000 individuals, which was over 100 times higher than the incidence rate in the general population. This emphasizes the strong association between HIV infection and the heightened risk of these severe reactions [2,3].

Cases with less than 10% body surface area (BSA) affected are categorized as SJS, whereas those with 10%-30% involvement are considered as an SJS/TEN overlap. Similarly, TEN is defined as cases where more than 30% of the BSA exhibits detachment [4].

The onset of SJS/TEN typically occurs within eight weeks following drug exposure, most commonly between four and 28 days after initiation [5]. Notably, the timing and presentation of cutaneous adverse drug reactions appear to be similar in both HIV-positive and HIV-negative individuals [6]. Initial symptoms are often non-specific and may precede the skin findings by several days in roughly one-third of the patients [1,2].

## Case presentation

A 36-year-old man presented with asthma treated as required with inhaled salbutamol, along with untreated allergic rhinitis since childhood, and mixed anxiety-depressive disorder diagnosed one year prior. He had been taking oral fluoxetine (20 mg/day) and carbamazepine (200 mg/day) for eight weeks. He reported no known drug allergies.

On May 19, 2025, he developed non-specific upper respiratory infection symptoms and self-administered a single oral dose of trimethoprim-sulfamethoxazole (TMP-SMX) (80/400 mg). Within six hours, he developed a maculopapular erythematous rash on his chest, accompanied by facial edema. The dermatosis then spread progressively to the posterior thorax, abdomen, and upper limbs. Subsequently, he developed a fever of 38.5°C and sought medical care, receiving antihistamine treatment. As his symptoms persisted, he presented to our medical center for further evaluation.

**Figure 1:**
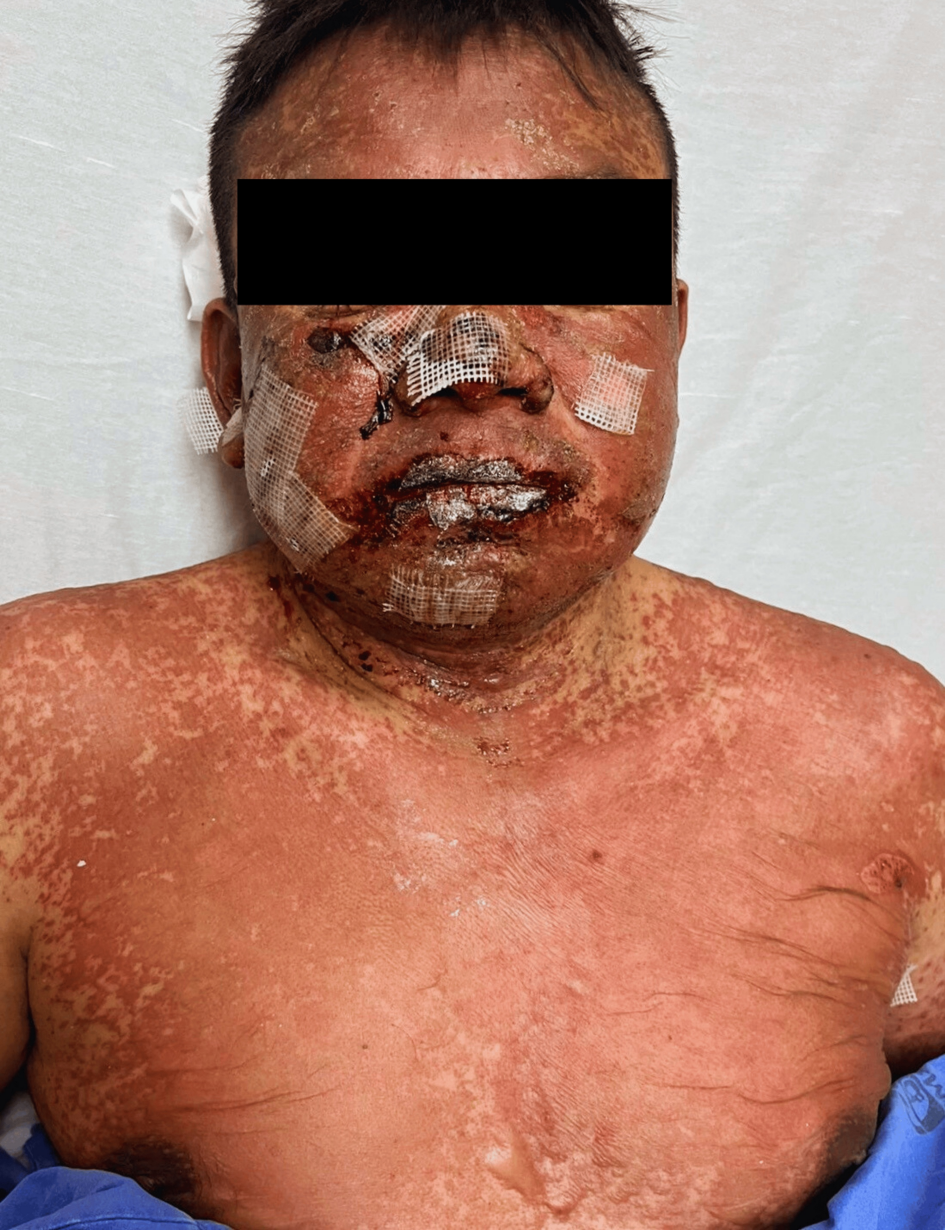
Cutaneous and mucosal involvement in TEN. Patient with dermatosis involving approximately 40% of the total body surface area. According to clinical classification, involvement exceeding 30% of the body surface area with epidermal detachment—or areas susceptible to detachment—is consistent with TEN. TEN: toxic epidermal necrolysis

**Figure 2:**
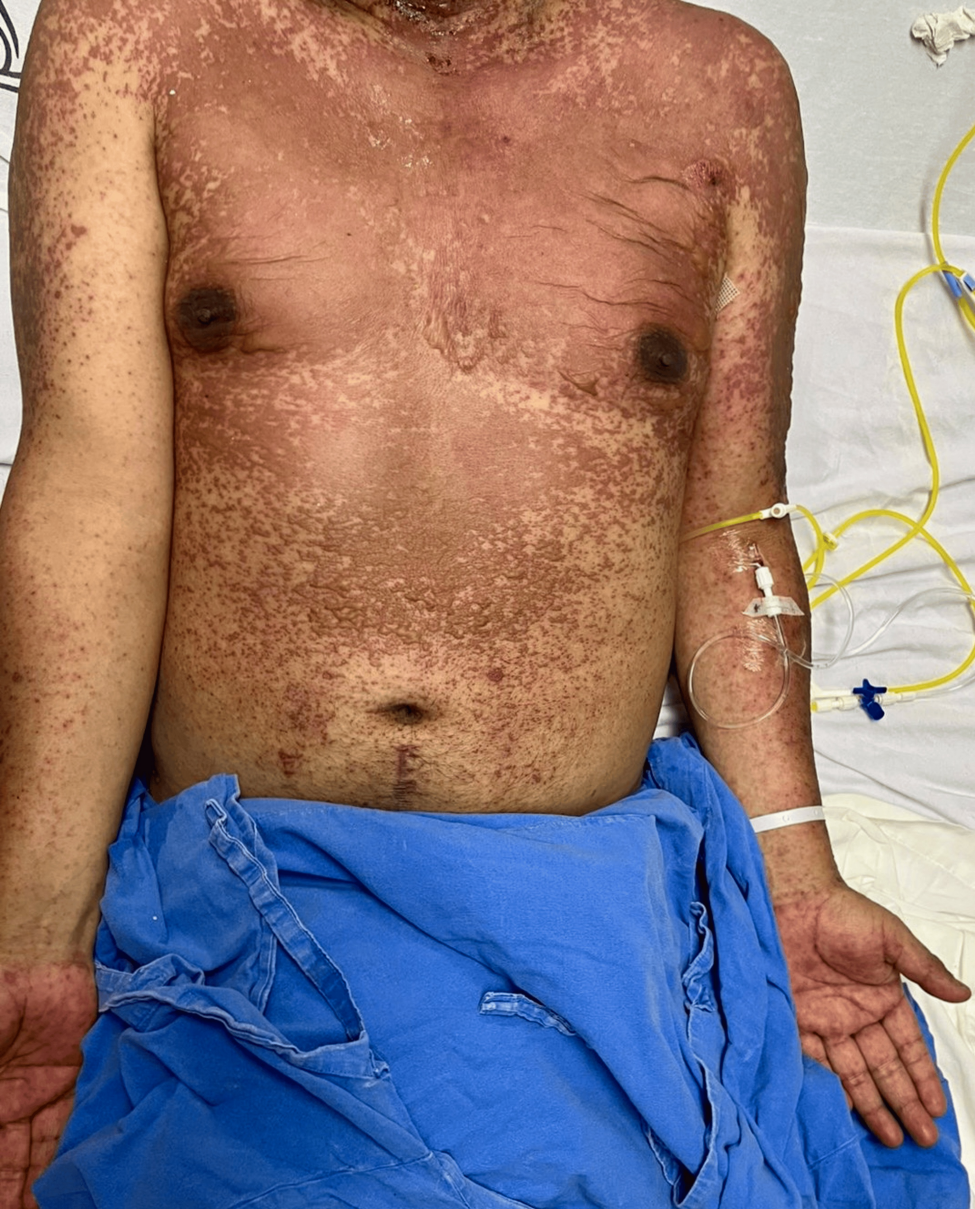
Dermatosis involving the chest and upper extremities.

Physical examinations revealed widespread dermatosis across all four body segments, with multiple poorly defined, confluent erythematous-violaceous macules topped by flaccid bullae containing serous fluid and erosions. The lips and oral mucosa exhibited erosions and necrotic areas covered by hemorrhagic crusts. Furthermore, serous discharge was observed from the ocular mucosa, and Nikolsky’s sign tested positive. The affected BSA was approximately 40% (Figures 1, 2).

Ophthalmological evaluation revealed abundant discharge at the base of the eyelashes, which impaired eyelid opening. The eyelids exhibited positive Nikolsky’s sign as well. The bulbar and tarsal conjunctivae were hyperemic, with the presence of pseudomembranes in the conjunctival fornices.

Upon admission, the patient was hemodynamically stable, afebrile, and without clinical evidence of sepsis or overt organ dysfunction. Laboratory tests at the time of admission revealed the following: glucose 89 mg/dL, urea 45.4 mg/dL, blood urea nitrogen (BUN) 21.2 mg/dL, creatinine 1.07 mg/dL, sodium 127.4 mmol/L, chloride 92.1 mmol/L, potassium 3.55 mmol/L, leukocytes 5.65 x 10⁹/L, hemoglobin 14.7 g/dL, hematocrit 41.5%, platelets 318 x 10⁹/L, absolute lymphocyte count 0.49 x 10⁹/L, monocytes 0.71 x 10⁹/L, eosinophils 0.06 x 10⁹/L, neutrophils 4.32 x 10⁹/L, and bicarbonate 18 mEq/L.

Based on clinical findings, a diagnosis of TEN was made, with a Severity-of-Illness Score for Toxic Epidermal Necrolysis (SCORTEN) score of 3. The patient declined to undergo a biopsy. He was admitted for inpatient care, which included systemic corticosteroids at a dose of 1 mg/kg/day (oral prednisone 75 mg/day) and a systemic calcineurin inhibitor (cyclosporine at 3 mg/kg/day), petrolatum-impregnated gauze dressings and reverse isolation, analgesia, parenteral fluid therapy, and nutritional support. All potentially implicated medications (carbamazepine and TMP-SMX) were discontinued. Ocular management comprised lubricants, ophthalmic tobramycin, and dexamethasone.

During hospitalization, a previously undiagnosed HIV infection was identified with a reactive test, exhibiting a viral load of 711,750 copies/mL and CD4+ count of 19 cells/µL. Opportunistic infections were ruled out, and antiretroviral therapy was subsequently initiated with bictegravir/tenofovir alafenamide/emtricitabine.

During the follow-up period, the patient demonstrated significant clinical improvement, presenting only residual post-inflammatory hyperpigmentation of the skin, with no evidence of visual acuity loss or other ocular sequelae. To date, the patient has not presented any post-treatment complications.

Notably, the patient was evaluated by the Allergy and Clinical Immunology department during his hospitalization. A detailed drug timeline was constructed. TMP-SMX had been previously tolerated six months earlier without adverse effects, and prior to admission, only a single dose was ingested. In contrast, carbamazepine had been commenced eight weeks (56 days) earlier at a fixed dose of 200 mg/day. The clinical course was deemed compatible with a type IV T cell-mediated hypersensitivity reaction. These reactions are characteristic of aromatic anticonvulsants such as carbamazepine and typically present with a latency of 6-8 weeks. Additionally, it was interpreted that the prodromal symptoms experienced on May 19 were likely part of the early immunological phase of the reaction.

Although TMP-SMX is one of the drugs most commonly associated with TEN and received a score of 4 on the Algorithm for Drug Causality in Epidermal Necrolysis (ALDEN) (indicating a “probable” association), its isolated administration and the rapid onset of symptoms following ingestion were not fully consistent with the expected immunopathologic mechanism. Therefore, the immunology team concluded that carbamazepine was the most likely causative agent, whereas TMP-SMX may have acted as a cofactor or exacerbated an already initiated immune response.

## Discussion

According to the consensus criteria established in 1993, the classification of SJS/TEN is based on the percentage of BSA affected by epidermal detachment. SJS involves less than 10% BSA, SJS/TEN overlap ranges from 10% to 30% BSA, and TEN is diagnosed when more than 30% of BSA is affected [4]. The distinction is specific to areas of skin detachment or susceptibility to detachment. Other types of lesions such as morbilliform rash, erythematous macules or patches, purpura, and target-like lesions on intact skin are not factored into this calculation. Due to the extensive involvement of the skin and mucous membranes, SJS/TEN can result in serious complications such as secondary infections, septicemia, multiple organ failure, and significant mortality. Consequently, the reported mortality rates may reach up to 34%-50% [1]. Furthermore, systemic involvement may occur through disruption of the epidermal barrier, ultimately leading to homeostatic imbalance, electrolyte disturbances, hypothermia, dehydration, or sepsis [1].

Initial laboratory testing should include a complete blood count and a comprehensive metabolic panel for the evaluation of electrolyte imbalance due to significant insensible fluid loss. Arterial blood gas analysis is also recommended to assess the respiratory and metabolic function. During hospitalization, daily monitoring of serum glucose, fluid balance, and electrolyte levels is essential in assisting quality supportive care [1].

Causality determination

Identifying the offending agent involves meticulous recording of the patient’s medical history and constructing a detailed drug timeline that includes all the prescribed medications. Information regarding the timing of drug initiation, discontinuation, dose adjustments, or temporary pauses is critical. Several tools have been developed to aid in this process, such as the Naranjo algorithm and the ALDEN score. ALDEN is considered the preferred method due to its disease-specific criteria tailored to SJS and TEN [1,7].

Prognostic assessment

Several scoring systems are available for predicting disease severity and mortality risk in patients with SJS/TEN. Of these, SCORTEN is the most widely validated and employs seven clinical variables: age > 40 years, active malignancy, heart rate > 120 bpm, serum BUN > 28 mg/dL, involvement of >10% BSA, serum bicarbonate < 20 mmol/L, and serum glucose > 250 mg/dL. It is recommended that SCORTEN be calculated on both day 1 and day 3 of hospitalization to estimate the mortality risk [1]. In this case, the SCORTEN score was calculated based on the patient’s clinical and laboratory data obtained on the day of admission (day 1). Each variable was assessed according to the established criteria: age, presence of malignancy, heart rate, BUN, BSA involvement, serum bicarbonate, and serum glucose.

Management

The management of SJS/TEN requires a comprehensive, multidisciplinary approach due to the potential risks of multiorgan involvement. Care teams should address wound and mucosal care, ophthalmologic and genitourinary care, pain control, airway protection, fluid and electrolyte replacement, nutritional support, prophylaxis for stress ulcers and thromboembolism, and prevention of nosocomial infections. Because sepsis is the leading cause of death in these patients, maintaining a sterile environment and practicing strict infection control measures are essential [1].

No universally accepted systemic therapy currently exists for SJS/TEN. Cyclosporine, which targets T cell activation, has demonstrated some potential in small observational studies by reducing the extent of skin detachment and the duration of hospital stay. Additionally, combined therapy with intravenous immunoglobulin and corticosteroids has been associated with decreased mortality [8,9].

Pathophysiology and HIV-associated immunity

Although the pathophysiological mechanisms behind TEN are not fully understood, it is widely accepted that T lymphocytes play a central role in inducing keratinocyte apoptosis, particularly through CD8+ cytotoxic T cells. On the other hand, regulatory T cells (Tregs), a subset of CD4+ cells expressing CD25, function to preserve immune tolerance and inhibit excessive cutaneous inflammation. Dysfunction or depletion of Tregs has been implicated in the pathogenesis of adverse cutaneous drug reactions, especially in immunosuppressed states like HIV. In individuals living with HIV, the increased vulnerability to severe drug hypersensitivity reactions is presumed to result from a complex interplay of immunologic, metabolic, viral, and host factors. Research has indicated that HIV-positive patients with TEN exhibit lower numbers of skin-homing CD4+ T cells, a higher CD8+/CD4+ ratio, and a reduction in CD4+CD25+ Tregs. This immunologic imbalance is believed to impair the regulation of cytotoxic responses, thereby facilitating the development of severe skin reactions to drugs [6,10,11].

## Conclusions

This case report highlights the importance of judicious use of systemic medication in patients, particularly in populations predisposed to severe adverse drug reactions. Careful evaluation of drug history combined with the awareness of individual risk factors, such as HIV infection and genetic predispositions, is essential to prevent life-threatening conditions like SJS and TEN. Moreover, multidisciplinary management and timely identification of the causative agent can improve patient outcomes and reduce mortality.

This case has some limitations. The absence of a skin biopsy limits histopathological confirmation of TEN. Additionally, the temporal overlap of TMP-SMX intake introduces some uncertainty about its role in triggering or exacerbating the reaction. Finally, longer-term follow-up data regarding immune recovery and potential sequelae are limited due to the timing of manuscript submission.
